# Book Reviews

**Published:** 1829-03

**Authors:** 


### CRITICAL ANALYSES.

Quæ laudanda forent, et quæ culpanda, vicissim

Illa, prius, cretâ; mox hæc, carbone, *notamus*.—Persius.

*Elements of General and Pathological Anatomy, adapted to the present State of Knowledge in that Science.* By David Craigie, M.D.—8vo. pp. 816. Adam Black, Edinburgh; Longman and Co. London. 1828.

Although we are by no means inclined, with some writers, to estimate the practical utility or skill of a physician by his knowledge of morbid anatomy, we are too sensible of the many important advantages that have been derived from a zealous attention to this department of medical science, to consider it as one that the practitioner can negligently pass over without compromising his own reputation, and endangering the safety of those who may be committed to his charge. It must, however, and it ought to be, confessed that he who may very correctly predict the remote effects of disease may be but very imperfectly acquainted with the best mode of arresting its progress while it is yet under the guidance of our art. It is equally true that many very important and destructive maladies depend upon functional derangement alone, and leave no physical traces which can be detected by the scalpel of the most accurate morbid pathologist. Highly important, then, as may be a perfect acquaintance with these alterations of structure which are caused by disease, we can neither consider morbid anatomy to be the most essential, or the first subject which ought to occupy the attention of the student, whose future life is to be devoted to the cure of disease. Let him first earnestly investigate the healthy actions of the system, then watch the primary symptoms and ordinary progress of disease; let him as carefully attend to the effects of the remedies he prescribes. When he has made himself master of these important subjects, he may apply himself to the study of morbid anatomy, and he will find it, at least, a very useful auxiliary to the information he has previously obtained. We cannot assign to this branch of our science a higher rank, and we consequently differ from those ancient and modern authorities who declare that it is the "basis of all medical skill." They give to morbid anatomy the station which physiology ought to occupy; for physiology must be considered the foundation of practical medicine.

The author of the volume before us is guided by this principle. We are led from the consideration of healthy to diseased structure, by concise yet perspicuous preliminary descriptions of the various organic tissues in their normal condition. This plan, in our opinion, very naturally enhances the value of the work.

Dr. CRAIGIE very truly observes, that the information which has been recorded upon the subject of morbid anatomy is scattered through so many volumes, that they can scarcely be consulted even in a cursory manner. So great is the accumulation of materials, yet so dispersed and multiplied, that the most intrepid diligence is disconcerted, and the most indefatigable perseverance exhausted.

"To alleviate, if not to remove, some of these difficulties, the most obvious plan is to classify the principal facts which it is important for the student to know; to reduce to general heads the numerous, isolated, and not unfrequently unarranged facts, recorded by different observers; to reconcile what is discordant; to explain what is anomalous; to distinguish the essential from the accidental, the important from the trivial; and to exhibit in a connected and systematic shape those deductions and inferences which are justified by accurate analytic comparison of the best authenticated facts. Though these are the objects which have been held in view in the composition of the present volume, it can only be determined by others with what success they have been attained." (*Preface*, p. vii.)

As the basis of arrangement, Dr. C. has chosen the distinctions of the component tissues of the animal body, as derived from the similitude and difference of their anatomical characters. The advantages of this method, he says, have been previously recognized by the high authorities of John Hunter, Carmichael Smith, Bichat, Dr. Thompson, and Beclard; but still no complete system of pathological anatomy has hitherto been constructed according to its principles. In describing the pathological changes incident to each tissue, it has been his study not so much to speak from personal observation, as to generalize with fidelity the results of the researches of others. In adducing the testimony of other observers, he has very prudently not run the risk of perpetuating error, by assuming as proved that which he himself has not had the opportunity of verifying. Of every morbid change described, the description is derived, in some instances, from repeated inspection; in all, from more or less personal examination of its physical and anatomical characters.

With a degree of sincerity which does not mark every prefatorial chapter, Dr. C. tells us what his book does not contain, as well as what it does.

"On one department of pathological anatomy, the reader will find little or no information in the present volume. I allude to local diseases, and to those varieties of malformation which consist in misapplications of the component parts of organs. These, it is almost superfluous to remark, cannot, without violation of the principles of arrangement, be introduced in a work on general anatomy; and I have, therefore, however reluctantly, excluded them almost entirely, unless so far as their general characters could be stated." (*Preface*, p. xii.)

On ordinary points, on which pathological opinion is unanimous, the author has been "sparing of reference, or omitted it entirely. The authorities referred to in illustration of various points of discussion, are selected from the most useful and most accessible."

It must evidently be impossible for us to enter into a deliberate consideration of every part of a work consisting of nearly a thousand pages. We can only convey to our readers an opinion of the whole by a selection of some of the most interesting parts.

Chapter i. *Division of the Textures.*—The progressive improvements that have been made by various distinguished writers on anatomy, are briefly mentioned. BICHAT is to a certain extent deprived of the laurels which have hitherto been yielded to him. Dr. Craigie states, that in the time of the elder HUNTER and CULLEN, general anatomy received very valuable contributions from an ingenious foreigner, ANDREW BONN, of Amsterdam. In his inaugural dissertation, contained in Sandifort's collection of Theses, which was published at Rotterdam in 1769, Bonn treats on the membranes and the structure of the skin; and, says our author, "it is an example of the capricious nature of scientific reputation, that, while the work of Bichat, which was published forty years after, though little more than the thesis of Bonn expanded, has given to its author an imperishable name, the small treatise of Bonn is equally unknown and unregarded, and has scarcely served to rescue his name from utter oblivion." (P. 11.) We confess we know nothing of Bonn's work. The statement of Dr. Craigie is doubtless correct. It is obvious, however, that the allegation of resemblance does not necessarily involve the charge of plagiary. Each probably drew from nature, and hence the similarity of the portraits they have presented.

In the brief view which our author takes of the organic tissues, he does not adhere entirely to either of the arrangements of previous pathological classifications. He modifies that of Bichat, which is perhaps the least objectionable, by adopting as many of the suggestions of his commentators as the nature of the subject and his personal observation may seem to authorize. The term "cellular membrane" is not correct in the opinion of Dr. C.: he proposes the adjective *filamentous*, as more appropriate. The filamentous tissue, then,

—" may be described as a substance consisting of very minute thready lines, which follow no uniform or invariable direction, but which, when gently raised by the forceps, present the appearance of a confused and irregular network. As these minute lines cross each other, they form between them spaces of a figure not easily determined, and perhaps not uniform. By some authors these spaces or intervals have been named cells; but, accurately speaking, the term is not fortunately applied. The component lines, which do not exceed the size of the silkworm threads, are so slender, that they do not form those distinct partitions which the term cell implies; and though, by forcible distention, such as takes place in insufflation or separation by forceps, cavities appear to be formed, these, it will be found, are artificial, and result from the separation of an infinity of the slender filaments of which the part is composed. These interlinear spaces necessarily communicate on every side with each other; and indeed the most distinct way of forming a true idea of the structure of the cellular tissue, is to suppose a certain space of the animal body which is divided and intersected into an infinite multitude of minute spaces (areolæ,) by slender thready lines crossing each other." (P. 24.)

In the adoption of this term, which he selected from personal observation and reflection, the author finds that he has been anticipated by C. B. de Bergen, whose anatomical accuracy he highly eulogizes.

If an incision is made into the filamentous tissue in the living body, it is found, if we except those fluids which issue from divided vessels, that nothing is observed to escape but a thin exhalation or vapour, which is evidently of an aqueous nature. By some authors this has been termed *cellular* serosity, from its resemblance to the serous part of the blood. Its quantity has been greatly exaggerated. In the living body it appears not to exist as a distinct fluid, but as a thin vapour, which communicates to the tissue the moist appearance which it possesses.

" This fluid is understood to be derived from the minute colourless capillaries named exhalants; and it is supposed to be no sooner poured forth in an insensible manner, than it is removed by the absorbing power either of lymphatics, according to the followers of the Hunterian hypothesis, or of minute veins, according to Magendie. It is of no great moment whether this process of absorption be ascribed to lymphatics or to veins, or be understood (as is probably the truth) to be effected by both. It is sufficient to remark, that whatever serous fluid is secreted into the interstitial spaces or cells of the filamentous tissue makes no long abode in that situation, but in the healthy state is speedily removed; so that, if we suppose exhalation, absorption must be also admitted; and the filamentous tissue is therefore represented as the seat of an incessant exhalation and absorption." (P. 26.)

It does not appear that the nervous twigs, observed to pass through the filamentous or cellular tissue, are lost in it. In general they have been traced to some contiguous part. The extensive distribution of the subcutaneous filamentous tissue, the mutual connexion of its parts, and its ready communication with the filamentous tissue of the mucous and serous membranes, have been demonstrated by Haller, W. Hunter, and Bordeu; and have been clearly explained by Portal and Bichat. The following principal points are worthy of attention:

"The filamentous tissue of the head and face communicate freely with each other, and with that of the brain by the cranial openings, and with the submucous tissue of the eyelids, nostrils, lips, and the inner surface of the mouth and cheeks. It communicates also with the subcutaneous tissue of the neck all round; and at the angle of the jaw, in the vicinity of the parotid gland, is the common point of reunion. To this anatomical fact is referred the frequency of swellings and purulent collections in the region of the parotid, in the course of various diseases of the head, face and neck.

"The filamentous tissue of the neck may be viewed as the connecting medium between that of the head and trunk. From the former region it may be traced downwards along the back, loins, breast, sides, flanks, and belly. At the cervical region, and between the shoulders, it is dense and abundant; and, surrounding the dorsal part of the vertebral column, it is connected with the mediastinal tissue, the submucous tissue of the lungs, and the subserous tissue of the costal pleura. At the fore part of the neck it is in like manner connected with the abundant tissue of the pectoral region, and by means of that surrounding the larynx and trachea, 1st, with the submucous tissue of the bronchi, and, 2d, with the anterior mediastinum. Passing downwards, the same communication may be traced with the intermuscular tissue of the loins and belly, the tissue surrounding the lumbar and sacral portion of the vertebral column, that connecting the mesentery and large vessels to the vertebræ, and extending all round under the muscular peritoneum, and into the pelvis, where, by means of the tissue at the posterior surface of the abdominal muscles, at the anterior surface of the iliacus internus, and through the obturator hole and ischiatic notch, it communicates with the filamentous tissue of the lower extremities. From the rectum and branches of the ischium it is continued along the perineum by the urethra, and into the scrotum.

" In the whole of this course it is abundant in the space before the vertebræ, round the psoæ and iliacus internus muscles, and round the bladder, rectum, prostate gland, and womb. The tissue surrounding the vertebral column communicates with that in the interior of the column by the intervertebral holes.

" The armpit may be considered as the point of union between the filamentous tissue of the trunk and that of the upper extremities, while the groin is the corresponding spot for the lower extremities. These facts should be kept in mind in observing the phenomena of diseases of this tissue.

" Notwithstanding this general connexion, however, certain parts of the tissue are so dense and close as to diminish greatly the facility of communication. Thus, along the median line it is so firm that air injected invariably stops, unless impelled by a force adequate to tear open its filaments; and water is rarely found effused in this situation. In the neighbourhood of some parts of the skeleton, also, as at the crest of the ilium, over the great trochanter, and on the shin, the filamentous tissue is very dense and coherent." (P. 30.)

The filamentous tissue is liable to inflammation, acute and chronic, circumscribed and with exudation of lymph, or diffused and spreading, generally without this exudation, and with the production of purulent matter; to induration; to hemorrhage; to serous infiltration; to aerial distention, and to new growths. Upon each of these morbid conditions Dr. C. offers many very instructive observations.

The adipose membrane which has frequently been confounded with the filamentous tissue, under the general name of cellular membrane, adipose membrane, and cellular fat, was distinguished and positively described by Beclard. Some obscurity hangs over the morbid alterations of this structure.

" In females and in eunuchs it is more abundant than in males. In females deprived of the ovaries it is more abundant than in those possessed of these organs; and it is well known that sterility is frequent among the corpulent of both sexes. In some circumstances this accumulation may be so great as to constitute disease, (Polysarcia adiposa; Cyrilli, Sauvages, Cullen, and Good;) and in other circumstances the deposition of fat is a means which the secreting system seems to employ to relieve fulness and tension of the vessels, and, if not to cure, at least to obviate morbid states of the circulation. (Parry.) Accumulations of fat are said to take place in some animals in a few hours in certain states of the atmosphere. During a fog of twenty-four hours' continuance, thrushes, wheatears, ortolans, and redbreasts, are reported to become so fat that they are unable to fly from the sportsman. (Bichat.)" (P. 63.)

The diminution or disappearance of fat is much more frequent than its extraordinary abundance, and depends upon various causes, which the author mentions. The removal of fat from its containing membrane is effected by the process of absorption, the agents of which some suppose to be the lymphatics; others ascribe it, at least in some measure, to the influence of minute veins.

" It is a point of some interest to know in what form it is absorbed, whether as oily matter, or after undergoing a process of decomposition. The observation of Dr. Traill would lead to the former view; but it is not easy to conceive that this should be uniform. We want, in short, correct facts on the point at issue." (P. 66.)

The adipose membrane is also the frequent seat of that singular change termed melanosis.

" The black or melanose matter is found in the subcutaneous adipose membrane and the subjacent cellular tissue of the chest and belly; it is not uncommon in the fat of the orbit; it is very commonly seen in the adipose cushion on the fore part of the vertebral column, that surrounding the kidneys, and in the fat of the anus and rectum; it is found in the anterior and posterior mediastinum; and it is found between the folds of the mesentery, of the mesocolon, and of the omentum. It is also found in the substance of the marrow of bones; and perhaps in most cases in which the osseous system appears to be stained with the melanose deposit, the dark matter may be traced to the medullary particles, the situation of which it is found accurately to occupy.

" In all these situations it appears in various degrees of perfection, and in different forms. It may be disseminated in black or inky spots through the adipose membrane; it may be accumulated in spherical or spheroidal masses of various size and shape; or it may be found in the form of brown or ebon-coloured fluid or semifluid, enclosed in a cyst formed of the contiguous tissue, more or less condensed.

" The melanose matter is entirely destitute of organization, and is to be regarded as the result of a peculiar secretion. No vessels have been traced into it; and, when bodies affected with this deposit are minutely injected, the vessels can be traced no farther than the enveloping cyst. (Breschet.) It is also to be noticed that it is never deposited exactly in the site of organic fibres, but always between them, and very generally in the precise situation of the adipose particles. These several circumstances show that the melanose disease consists not in a degeneration or conversion into another substance, but in the deposition of a new form of matter, in the manner of a secretion.

"In what form the melanose substance is first deposited, we have few accurate facts to enable us to form a judgment. Laennec is of opinion that it is first deposited in a solid form, and afterwards becomes fluid. The former he considers the stage of crudity, the latter that of softening (*ramollissement*). Several facts, however, would lead to the conclusion, that when first deposited it was fluid, and afterwards acquired consistency. Thus, in several dissections performed by Dr. Cullen and Mr. Carsewell, the matter of the small tumors, which are supposed to be of short duration, were found to be softest, and sometimes as fluid as cream. In like manner, in a case recorded by M. Chomel, in which the disease was found in the liver in the shape of large cysts, the melanose matter was more fluid in the centre than in the circumference of the cysts. Upon the whole, if the melanose deposit be, as is supposed, an inorganic secretion, the idea of its being poured forth from the vessels at first in a fluid or semifluid state is most probable, and most consistent with the usual phenomena and laws of animal processes." (P. 68.)*

In Chapter iv. we have a good description of the structure of arterial tissue. Dr. Craigie has repeatedly examined almost every considerable artery of the human body, and has never been able to recognize any longitudinal fibres either in the middle or proper coat, or in their internal membrane, as taught by Willis, Douglas, and De la Sône. It is said by Bichat, that the arteries derive their nerves almost exclusively from the ganglions and the gangliar nerves.

"The inference does not rest upon strict observation, and evidently owes its birth to the hypothetical opinions of this ingenious physiologist. All the arteries going to the extremities, the axillary and iliac, and their branches, receive nerves from the neighbouring nervous trunks, which are formed chiefly from cerebral or spinal nerves, and have no immediate connexion with the system of the ganglions. In the internal carotid and the vertebral arteries, and their branches, nerves cannot be distinctly traced." (P. 79.

To ascertain the several modes in which arteries terminate, has always been a problem of much interest to the physiologist, and great difficulty to the anatomist.

"1. The first undoubted termination of arteries is immediately in veins. It is unnecessary to adduce in support of this fact the long list of observers enumerated by Haller. It is sufficient to say that it was clearly established by the microscopical observations of Leuwenhoeck, Cowper, and Baker, by Haller himself, and

* The 32d volume of the Dict. des Sciences Medicales, page 183, contain a brief yet interesting account of this morbid change.—Rev.

by Spallanzani in his beautiful experiments on the circulation of the blood.

"2. The second termination which may be mentioned here is that into the colourless artery, (*arteria non rubra.)* This is sufficiently well established by the phenomena of injections.

"3. A third termination which is supposed to exist, but of which no sensible proofs can be given, is that into colourless vessels supposed to open by minute orifices on various membranous surfaces, and therefore termed exhalants." (P. 85.)

The nature of the exhalants is subsequently considered.

HALLER admits a termination in, or communication with lymphatic vessels, but allows that it is highly problematical.

In describing the alterations produced by disease in the arterial structure, Dr. C. remarks that "a red or crimson staining of the inner membrane, especially in the aorta, has been mentioned by Corvisart, Frank, Hodgson, and Laennec, and may be often seen in persons who have died without symptoms of pectoral or arterial disorder. Its nature is not well known. It seems to be the effect of a dying or tinging property of the blood, either during the last moments of life, or after the heart has ceased to beat. It must not be confounded with inflammation or its effects." (P. 89.)

The subject of aneurism is too important to have been so cursorily touched upon as it is by our author.

The nature of the calcareous depositions to which arteries are liable has given rise to various speculations.

"But this variance has partly arisen from the practice of confounding it with the steatomatous deposition. It is said to differ from osseous matter in two circumstances: First, the deposition is earthy from the first, without any previous matrix of animal matter; secondly, it is destitute of the usual fibrous structure, and presents an irregular but homogeneous crust without any obvious arrangement. It consists, however, of the usual combination of animal matter and bone earth. A specimen analyzed by Mr. Brand gave 65.5 parts of phosphate of lime, and 34.5 of animal matter in the 100 parts. The latter was chiefly albumen, with traces of gelatine." (P. 93.)

It has been said by SCARPA that the steatomatous deposition in the arterial tissue proceeds invariably to ulceration. Dr. Craigie, however, assures us that this is not an invariable result; for a large portion of an artery may be affected with it without suffering the smallest breach of continuity or destruction of tissue.

The ensuing chapter treats of the venous tissue. We must pass over the anatomical description of veins. It is worthy of observation that Bichat has remarked that the osseous or calcareous depositions, which are common in various spots of the inner arterial membrane, and especially at the mitral and aortic valves, are never found in the inner venous membrane, or at the tricuspid valve, or in the semilunar valves of the pulmonary artery. Dr. Craigie suggests, as a question, whether these depositions have been found inside the pulmonary veins, and not inside the pulmonary artery?

Venous tissue is liable to inflammation, adhesive or circumscribed, and spreading, generally suppurative; to *varix*, to osseous deposition, and to the formation of concretions. The subject of spreading inflammation of the venous tissue is passed over much too briefly. The dangerous nature of this affection of the veins, and the many highly interesting points connected with it, which still require investigation, claim the most serious attention.*

The *system of capillary vessels*, *terminations of arteries*, and *origins of veins*, are described in the next chapter. The author very correctly states that, although the term "capillary system" is constantly used, it is neither precisely defined nor distinctly understood. According to Bichat, it is not only the common intermediate system between the arteries and veins, but the origin of all the exhalant and excreting vessels. To this doctrine Dr. Craigie replies, that "if we consider the modes in which arteries have been said to terminate, and veins to take their origin, we shall find that, in this view of the capillary system, there are some things which are doubtful, and some which are inconsistent with the rest."

"Of the different kinds of terminations assigned to arteries, and of origins assigned to veins, one only admits of sensible and satisfactory demonstration. Arteries, when they have so much diminished as to become capillary, are seen by the microscope, in some instances by the naked eye, to pass directly into corresponding capillary veins, or to end abruptly in some organ or membrane unconnected with any other vessel. It is likewise certain that the microscope shows every capillary vein to arise from a capillary artery; and, if there be any other mode of origin, it has not yet been demonstrated or established. Only one other circumstance

* Mr. Arnott, in the very excellent paper which he lately presented to the Medical and Chirurgical Society, has, we think, very satisfactorily shown that some of the doctrines admitted by Dr. Craigie, and indeed generally acknowledged, are inaccurate. Mr. Arnott's communication will, of course, be published; and we shall not fail to convey to our readers the original opinions he has derived from a most industrious and judicious selection of facts.—Rev.

requires to be taken into account in this inquiry. This is, that the capillary artery and vein may contain either red or colourless blood; for, according to the size of the vessels, and the nature of the organs or tissues in which they are distributed, the blood which flows through them will be coloured or colourless. This view of the communication of minute arteries and veins, which is perfectly consistent with the known facts, will afford the only explanation which it is possible to give of the singular division of the capillary system which Bichat has chosen." (P. 136.)

There is no precise point at which the arterial tissue or structure can be said to terminate, and none at which the venous structure can be said to commence. By some a direct communication of minute arteries and veins is denied. The organic properties of the capillary vessels are as little known as their structure. Many physiological and pathological writers, especially experimentalists, have ascribed to them a power which has at different times been called muscular, tonic, irritable, contractile; and have asserted, Dr. Craigie remarks, " that, because the larger arteries are provided with a fibrous membrane, which they have called muscular, and to which they have ascribed irritability, or the power of contraction when stimulated, their minute or capillary terminations must have the same property. This conclusion is completely unfounded, for two reasons: First, I have already shown that the proper arterial tunic is not muscular in structure, and, according to the best experiments, possesses no property of contraction when stimulated. Second, although it be admitted that the proper arterial tissue is muscular and irritable, it is quite certain that observation has not hitherto shown that this tunic can be recognized in arteries smaller than a line in diameter; and it is certain that in the capillaries, properly so called, (that is, in the vessels which partake of the nature of artery and vein,) no such structure has yet been observed." (P. 140.)

We are not acquainted with any physiological or pathological writers who have *thus* assumed the irritability of the capillaries. The evidence upon this subject is not presumptive. That the capillary vessels are endowed with the only kind of vital action with which we are acquainted, namely contractility, has been demonstrated by the experiments of Hastings and Wilson Philip. It would be easy to adduce the authority of other experimentalists. To accumulate proofs, however, must be unnecessary; for the experiments of Hastings, to which alone we have an opportunity of referring, are not to be resisted.* Dr. Craigie is of opinion that the conclusion drawn from these experiments, as well as from those of HUNTER and THOMPSON, is not satisfactory.

"The effects which the application of mechanical irritants, or chemical substances, as alcohol, acids, and alkalis, produced in the experiments of Hunter, Wilson Philip, Thomson, and Hastings, have been supposed to demonstrate the irritable nature of the capillary vessels. The conclusion is illegitimate, in so far as the results of these experiments are open to several sources of fallacy. In some instances these effects are to be ascribed to incipient inflammation; in others, to shrivelling of the capillary structure, or crispation by chemical action; in others, to actual coagulation of the blood of the capillaries; but none of them prove satisfactorily or precisely any peculiar properties in the vessels of which the capillary system is composed." (P. 142.)

In our opinion, the experiments referred to prove to conviction the point in dispute. We do not conceive, with Dr. Craigie, that the results are either equivocal or uncertain. It signifies but little, or indeed nothing, for the argument, whether the coats of arteries are muscular in structure or not. We do not deem it necessary to enter upon this oft-disputed and still-disputable point. That the blood vessels are endowed with a vital power of contractility, resembling that of the muscles, and therefore called muscular, we think is clearly established. The term *tonicity*, suggested by Parry, is perhaps preferable, as it is less likely to perpetuate a mere verbal dispute. Dr. Craigie appears to have looked for muscular structure where he was least likely to detect it, viz. in the *considerable* arteries of the human body. The irritability of arteries is in an inverse ratio to their size, and consequently the existence of this property should be sought for in the small or capillary vessels.

The morbid deviations incident to the system of capillary vessels are of the utmost importance. They are the main agents of most of the healthy processes of the animal body, and there are few morbid states in which their operation is not primary, or of which they do not more or less partake. Numerous opponents may be found to the doctrine conveyed in the following passage: we think it is correct.

"Upon the whole, two facts may be considered to be established regarding the state of the capillary vessels of an inflamed part.

* An account of these experiments is prefixed to Dr. HASTINGS' excellent treatise "on Inflammation of the Mucous Membranes of the Lungs."

The first is, that these vessels are unnaturally and unusually distended, and really contain more blood than in the state of health. This is proved not only by incisions into inflamed parts, but by dissections of every part and organ of the body. The second is, that the blood moves more slowly in these vessels than in the healthy state, and even after some time may remain entirely motionless. This is also established by observing the effects of inflammation in the human body, but especially by the phenomena of inflammation excited artificially in the bodies of the lower animals." (P. 147.)

It is still a point to be ascertained, whether these two conditions constitute the essence of inflammation. It is equally undetermined by what agency these states are induced in the capillary system of any tissue or organ.

We cannot dwell upon the description given of the various morbid conditions of the capillary vessels, although it is worthy of attentive perusal.

In the second section of the thirteenth chapter, we approach a very important part of pathological study, namely, *diseased conditions of the brain.* It is mortifying, but it is very true, that, notwithstanding the vast labour that has been expended upon the anatomy and pathology of this organ, we possess but few positive facts relative to either, which can instruct us as to the origin of its diseases, the symptoms which different lesions produce, or the treatment which they may require. Knowledge which is worth acquiring is rarely obtained without determined perseverance: we must not, therefore, abate our diligence because it has been as yet but scantily rewarded.

Dr. Craigie differs from the opinion of Dr. ABERCROMBIE and other pathologists respecting *ramollissement* of the brain. He does not conceive that this condition is of the nature of gangrene in other parts. "A part of the brain changed as above described is indeed disorganized, may be said to be dead, and in this sense the change may be termed *gangrene of the brain.* But when it is found in different degrees, and in so many different morbid states of the brain, some of them of long continuance, it is difficult to be satisfied that every one of them must be viewed equally as gangrene." (P. 383.)

The effects of this disease on the system are not very well distinguished.

"They may be divided into common and proper. The common effects are dull pain, or sense of weight in the head, dulness, impaired memory, frequent drowsiness, and occasional peevishness at trifles, and paralytic affections of the face, head, and members. The proper effects are sense of formication, numbness, and rigidity, or occasional involuntary contractions of the muscles of the upper extremities, followed by delirium or fatuity, and a peculiar odour about the head, not dissimilar to that of the mouse. In the spinal cord it gives rise to numbness and rigid contraction of the muscles of the lower extremities, and eventually palsy, more or less complete.

"These symptoms, which are chiefly those given by the French authors already mentioned, apply to the acute form of the disease. In more chronic states, it seems not to affect the muscular motions considerably, but rather to induce fatuity, and other forms of impaired intellect. This inference at least results from some of the observations of Morgagni, and those of Mr. John Hunter. This is the state of brain which takes place in cases of fatuity succeeding coup-de-soleil." (P. 384.)

Suppurative inflammation frequently takes place in various parts of the brain. One variety of cerebral abscess is connected with discharge from the ear. "Purulent discharge from the ear-hole is indeed generally connected with inflammation, subacute or chronic, of the dura mater, or vascular membrane, or both; and in some instances the disease takes an unfavorable turn in this manner, and speedily proceeds to a fatal termination." We would alter the word "generally" in this passage for "sometimes." In children, particularly, we frequently see discharges of pus from the ear, especially if there is any eruptive disease upon the scalp, when their health does not materially suffer either during the progress, or at the subsidence, or cure of the cutaneous malady. In such cases we cannot suppose that inflammation of the meninges of the brain has existed.

Dr. Craigie conceives it legitimate to infer that the essential anatomical character of apoplexy is injection of the vessels of the brain, more or less general. This may terminate in one of two modes, both of which are accidental and accessory. The first is effusion of serous fluid; the second is effusion of red blood. "If from any cause the circulation within the head becomes unusually slow, and the vessels of the brain become inordinately distended, either red blood or serous fluid is poured out from the extremities of the arteries. The latter process, if we admit the testimony of Cheselden, Morgagni, and Willan, takes place in the slow and gradual drowsiness and stupefaction which distinguish the form of the disease termed lethargy, (*veternus.*) The cause of the symptoms, however, is not the effusion, as Willan imagined, but the general vascular distention and injection from which the effusion arises." (P. 402.)

The state of the cerebral vessels which terminates in hemorrhage may occur, perhaps, at any period of adult life. But these blood-vessels are liable to a peculiar state which predisposes to extravasation.

"This consists in deposition of earthy matter between the coats of the internal carotid arteries and of the basilar artery, and their branches. In consequence of this deposition, they lose part of their contractile and distensile powers, and some of their tenacity, and whenever blood is accumulated in unusual quantity, as they do not so readily admit of distention, rupture is the consequence. (Baillie, 454.) Hodgson also shows how generally this morbid state of the cerebal arteries is connected with extravasation.

"This cause, however, is predisponent only. A fit of apoplexy may occur, and prove fatal, in persons in whom neither ossification of the arteries of the brain, nor any other state, except mere vascular injection, is found. And, on the other hand, the cerebral arteries may be ossified or steatomatous in many persons who have never had a single fit of apoplexy. The general result of the cases observed by Vater, Morgagni, Cheyne, Howship, Rochoux, Serres, and Tacheron, is, that disease of the arterial coats is connected with vascular injection, which may terminate, according to circumstances, in serous effusion, pulpy destruction, or bloody extravasation. It is a well-established fact, however, that the extravasation does not take place from the diseased arterial trunks, but from the minute capillaries in which these arteries terminate." (P. 412.)

Old age is attended with two circumstances which predispose to apoplexy. The first is the venous plethora so ingeniously maintained by Cullen. The second is the tendency which the arterial system more especially betrays to become diseased after the meridian of life. According to the present state of evidence, the author thinks it wisest to adopt the opinion of those who do not recognize that species of apoplexy to which the term "nervous" has been applied.

Palsy is supposed to depend on the same state of the cerebral capillaries which causes the general apoplectic affection, which it either precedes, accompanies, or follows; or on that state of the brain or spinal cord which terminates in pulpy destruction. In attempting to establish clearly the anatomical characters of palsy, two circumstances merit particular attention.

"First, several cases of apoplectic death are preceded by paralytic affection of one side, more or less extensive, in the successive forms of distortion of one side of the face, loss of speech, loss of power in an arm, a leg, or the entire side. When these phenomena are followed by coma and death, necroscopic inspection shows, as in apoplexy, capillary injection, with or without extravasation, and generally more or less destruction of brain. The commencement of the morbid process in this instance is doubtless the same capillary injection of part of the organ which, in a more exquisite degree, produces the comatose state. Secondly, though there are not a few instances in which an attack of loss of consciousness, sensation, and motion, is not followed by loss of voluntary motion, these, I have already attempted to show, depend on that capillary injection which is removeable by the use of remedies. When the capillary injection proceeds to destruction, either by hemorrhage, by softening, or by ulceration, (i. e. by superficial pulpy destruction of cerebral substance, consequent on hemorrhage,) it almost invariably leaves after it more or less of voluntary motion, generally on the side of the body opposite to that of the brain which has sustained the lesion. (K.) One of the most frequent effects of cerebral hemorrhage and its consequences, indeed, is palsy of the hemiplegic form; and in the brains of such persons as have laboured under this disease, either a broken-down and softened spot, or one or more hemorrhagic cavities or cysts, are found after death. (Wepfer, Willis, Morgagni, John Hunter, Baillie, Wilson, Abernethy, Rochoux, Serres, Lerminier, Tacheron, Abercrombie.) The general accuracy of these conclusions is confirmed not only by the necroscopic appearances of the brains of those who die of coma succeeding to palsy, but of those who die of the effects of injuries of the head, of abscess of the brain and cerebellum, and of tumors and other organic changes taking place either in the brain or in its membranes." (P. 421.)

The effects produced by capillary injection and hemorrhage in the spinal cord vary according to the stage of the process, and the region of the cord in which it occurs. "In the stage of injection it produces irregular involuntary twitches of the muscles of the trunk and extremities, numbness and coldness of the skin about the back, and occasionally of the limbs, and more or less loss of muscular power. In the advanced stage, whether that of hemorrhage or of pulpy destruction, numbness and palsy of the paraplegic form are complete." (P. 427.)

In some instances the state of capillary injection appears to give rise to tetanic symptoms. It is stated that in pulmonary consumption, whether depending on chronic bronchial inflammation, chronic pleurisy, or on tubercular disorganization, the brain is invariably found softer than natural.

"When the disease which induces death has continued long, this softness is very considerable, and amounts almost to semi-fluidity. It may then constitute a true cause of adventitious disease. This state of the brain, combined with a languid and retarded motion of the blood through the cerebral capillaries, may be the pathological cause of the delirium, which, either alone or alternating with coma, not unfrequently precedes the death of phthisical patients." (P. 435.)

Confinement, with inactivity and low diet, tend to impair the firmness of the brain.

Induration of the brain is occasionally met with. The cause of this change, and the means by which it is affected, are not known. Various other morbid conditions of the brain are described by the author.

In the following chapters the anatomy and pathology of the following structures are considered: Muscle, sinew, white fibrous system, yellow fibrous system, bone, gristle, fibro-cartilage, skin, mucous, serous, and synovial membrane.

We have paid very deliberate attention to this volume, and have formed a very favorable opinion of its merit. He who is yet to learn will obtain from it much excellent elementary information, conveyed in a very perspicuous form; and he from whose memory may be gradually escaping knowledge once possessed, will find Dr. Craigie's work a convenient and valuable reference, in which extensive research is displayed without unnecessary pomp of learning.

We purposely deferred offering any comment upon the arrangement adopted by Dr. Craigie, until we had seen how it would maintain the general purposes of the proposed investigation. Although the arrangement of the diseased conditions of different structures according to their anatomical distinctions is much too artificial to be strictly correct, it is still sufficiently so, if the pathological inquirer bears in mind that disease is seldom confined to one structure alone. Inflammation, as it has been observed by Thomson, in his excellent "Lectures on Inflammation," in all the different textures and organs of the body, may, in one point of view, be regarded as forming one and the same specific disease, although modified by texture. The progress and duration of disease in general is also as much, or more, modified by its original cause, and the general constitution and diathesis of the person affected, than by the difference in the texture of the part in which it occurs.

Carmichael Smith, Pinel, and Bichat, have adopted the principle of pathological arrangement which is acted upon in the present work. Dr. Craigie observes, in his Preface, "that the advantages of this method have been recognized by John Hunter." We confess we thought Hunter was opposed to the doctrines of Carmichael Smith and others upon this subject: he states, "It has been supposed that the different species or varieties of inflammation arise from the difference in the nature of the part inflamed; *but this is certainly not the case*, for, if it was, we should soon be made acquainted with all the different inflammations in the same person, at the same time, and even in the same wound. For instance, in an amputation of a leg, where we cut through the skin, cellular membrane, muscle, tendon, periosteum, bone, and marrow, the skin should give us the inflammation of its kind, the cellular membrane of its kind, the muscles of theirs, the periosteum, bone, marrow, &c. of theirs; but we find it is the same inflammation in them all."*

---

*A Letter to the Right Hon. the Secretary of State for the Home Department, containing Remarks on the Report of the Select Committee of the House of Commons on Anatomy, and pointing out the Means by which the Science may be cultivated with advantage and safety to the Public.* By G. J. GUTHRIE, F.R.S., Professor of Anatomy and Surgery to the Royal College of Surgeons; Surgeon to the Westminster Hospital, and to the Royal Westminster Ophthalmic Hospital, &c.—8vo. Sams, London, 1829.

It is unnecessary to enter into formal arguments upon points which excite no difference of opinion. The practice of dissection is admitted to be absolutely necessary: and it is equally admitted that the mode in which the anatomical schools have hitherto derived a supply of bodies, in this country, is highly disgraceful. We shudder when we reflect upon the recent proofs which have been afforded that it is also highly dangerous. It is surely time that our legislators should show, by their active and immediate interference, that they are neither totally indifferent to the cause of science, or deaf to the appeals of the public. We have always been convinced that the difficulties which surround this subject are more imaginary than real; and we are inclined to believe that it is not of very great importance what precise regulations the government may lay down to legalize the supply of subjects for dissection, provided they throw open some of the numerous sources from which bodies may be procured, under proper restrictions, and thus rescue the anatomist from the detestable necessity of holding communion with a horde of wretches, who are unworthy the title of men.

The plan suggested by Mr. GUTHRIE, in the very elo-

* Hunter on Inflammation, vol. i. p. 473, Travers's edition.

quent letter before us, might, we think, be improved in some of its details. He recommends the supply of dead bodies for public dissection to be derived from the following sources:

"1. All persons hanged, or otherwise executed, and for all offences whatsoever.

"2. All persons who die under sentence for criminal offences, whether in the hulks, gaol, penitentiary, or elsewhere.

"3. All persons who die in temporary or floating hospitals, in gaol, penitentiary, or other place of detention, or prison, from whatever cause they may have been placed there, and who have no friends to bury them.

"4. All persons found dead, from whatever causes, in highways, canals, or otherwise, and who, having no friends to bury them, are sent to bone-houses for interment, at the expense of the parish or county.

"5, and lastly. The poor who die in workhouses, having no friends to bury them, having expressed no wish on the subject, and having no respectable or decent relatives to express it for them, either before or after death.

"It is not proposed to interfere by regulations with the bodies of those who die without friends in regularly established hospitals; it being presumed that the surgeons of those institutions will properly apply them in the instruction of the students committed to their charge. In other words, it is not intended that the public schools of anatomy shall interfere with the private or public instruction delivered by surgeons in their own hospitals.

"The means of supply being furnished, the following regulations are proposed, to ensure a fair and regular distribution, which must be enforced, in one way or other, by legal enactment. It being understood that there are no laws on the subject to repeal, save that one, or part of one, which directs murderers to be hanged until dead, 'and their bodies to be given over for dissection,' and for the reception of which bodies the College of Surgeons is bound by their charter to find a proper place; which is at present in the vicinity of Newgate.

"*Laws proposed to be enacted.*—1. Punishing all persons actually engaged in exhuming or stealing a dead body, or of selling it without authority, and who can be proved to be so engaged after this session of Parliament. For the first offence, six months to hard labour, and to find two securities, in fifty pounds each, for future good behaviour; to be kept to hard labour until procured. For the second offence, double the punishment. Medical or other persons knowingly receiving such dead bodies, three months to the treadmill, and a fine of one hundred pounds; to be kept to hard labour until paid.

"Rendering the practice of dissection, and the possessing of dead bodies, legal; and protecting the persons so employed, and their property, by the same laws as protect persons and property generally.

"3. Directing the five sources of supply of dead bodies, as at pages 30 and 31.

"4. Declaring it to be illegal to require, or to take during life, in any hospital, workhouse, or other place for the reception of sick or poor people, securities in money or otherwise for the burial of such persons. Penalty, twenty pounds.

"5. Declaring it legal, and directing all treasurers, governors, trustees, or others in authority, in hospitals or other places; and all vestries, church-wardens, overseers of the poor, and others in authority in the parishes, to give over for dissection to the College of Surgeons, or persons appointed by them, the bodies of all persons who have died under their care or charge, without the means of burying them, and who have no relatives, or persons previously known to have been friends, who are willing to do it; and all other bodies in their charge which come within the meaning of classes 4 and 5 of the means of supply indicated, pages 30 and 31.

"6. Appointing the Royal College of Surgeons of London, by their secretary, or other person nominated by them, the proper authority or authorities to whose order the bodies are to be delivered.

"7. The Royal College of Surgeons to report quarterly, to the Secretary of State for the Home Department, on every point connected with this subject.

"8. The funeral service to be read over all bodies (unless forbidden by law,) before delivery for dissection.

"9. Legalizing the sale of a dead body by the friends of the deceased, after it has been viewed in the usual manner by the parish or other authorities.

"10. The Council of the Royal College of Surgeons, in making regulations for the proper distribution of the bodies placed at their disposal, to find a proper cemetery in various parish churchyards for the interment of remains after dissection; and the Council of the College to be authorized to make such charge for each body as may be considered proper; subject to the approval of the Secretary of State for the Home Department.

"11. All minor regulations of arrangement and detail made by the Council of the Royal College of Surgeons, and approved by the Secretary of State for the Home Department, to be binding on the different persons concerned. Penalty, twenty pounds.

"12. Every dispute which may occur, and every offence to which a penalty is attached, to be settled by information laid in the usual manner, before any three police magistrates of the division in which the offence has been committed; and whose decision shall be final.

"In order to enable all parties to act with precision and a due regard to decorum, the following minor arrangements are proposed, under the authority of the Secretary of State, to be varied from time to time, by his sanction.

"The Council of the Royal College of Surgeons, having the collection and distribution of all dead bodies intended for dissection, directs,

"1. An establishment of men, four or eight in number, to be ready for service every evening in the winter season from six to ten o'clock, and to proceed as directed with a shell (in a manner similar to that at present adopted by undertakers,) to the spot where the body is to be found.

"2. An establishment of one or two plain hearses, with two horses, a driver, and an attendant, in black, (like an undertaker's party,) to be ready to go to greater distances.

"3. The secretary, or proper officer appointed by the College, gives an order for the delivery of the body, which will be the receipt to the person who delivers it.

"The servant of the College who receives the body, delivers it again, according to an order received to that effect from the secretary; and the anatomist or gentleman who receives the body from him gives an acknowledgment, signed by himself or his assistant.

"In order to enable the secretary of the College to act with the necessary precision, the keepers of gaols, hulks, and penitentiaries, or other prisons, and the masters or governors of workhouses, and temporary or floating hospitals, should be directed, under certain penalties, to inform the secretary, or officer appointed by the College, when a person dies who is, by the preceding laws, ordered to be given over for dissection; and it will be the duty of the secretary, or other officer of the College, to signify in return at what hour the body will be sent for. Printed forms of communication to be furnished by the College, and letters (all paid by the College) to be sent within twenty-four hours.

"Teachers of anatomy to transmit every Monday morning to the secretary, or proper officer of the College, a return of the number of students wishing to dissect, and of the probable number of bodies required during the week; at the same time, a return of the number received during the past week, and the sum due for them at the price fixed. Practitioners, not being teachers, wishing to have a body for dissection, to communicate in a similar manner, their request being submitted for the approval of the president or vice-presidents."

The anatomical professors would, we think, very naturally object to the enactment of a law which would place them so completely at the mercy of the College of Surgeons. As the Council of the College is at present constituted, no apprehension either of partiality or illiberality is to be feared. But who can look into futurity, and say that it shall always be guided by the same principles of integrity and impartiality. Some anatomical teachers may not be admitted to a seat in the Council, while others might enjoy that honour. In case there should be a scarcity of subjects for dissection, it is more than probable that the claims of the latter would be preferred, and hence would immediately arise jarring and discontent. In many points of view we deem it objectionable that any one part of the profession should be invested with the distribution of bodies, for the use of those to whom they may be professionally opposed. We are not inclined to judge harshly of human nature, but we foresee the great probability of discord springing up amongst us, and of constant evasions of any laws that may be enacted, if it should be rendered necessary to apply to the Council of the College for every body that may be required for dissection.

The appeal that Mr. Guthrie makes to the Secretary of State in this letter, will doubtless meet with attention. To one part of the plan we have ventured to object. With some modifications, however, it appears to us capable of effecting the desired object of supplying the anatomical student with subjects for dissection, without wounding the feelings of the public. It must not be imagined that any plan can be devised which will not have to encounter temporary opposition. The clamor may be loud at first, but it need not be feared that it will last long.

---

### COLLECTANEA.

Floriferis ut apes in saltibus omnia libant,
Omnia nos, itidem, depascimur aurea dicta.

