# Erratum in the Last Number

**Published:** 1829-04

**Authors:** 


ERRATUM *in the last Number.* P. 244, *five lines from top of the page, for* "naturally" *read* "materially."

